# A training intervention on child feeding among primary healthcare workers in Ibadan Municipality

**DOI:** 10.4102/phcfm.v8i1.884

**Published:** 2016-09-20

**Authors:** Folake O. Samuel, Funmilola M. Olaolorun, Joshua D. Adeniyi

**Affiliations:** 1 Department of Human Nutrition, University of Ibadan, Ibadan, Nigeria; 2 Department of Community Medicine, University of Ibadan, Ibadan, Nigeria; 3 Department of Health Promotion and Education, University of Ibadan, Ibadan, Nigeria

## Abstract

**Introduction:**

Health workers at the primary level are well positioned to provide health information and counselling on child feeding to mothers on antenatal visits. The study was designed to evaluate the effect of training on the knowledge, attitudes and provision of infant and young child feeding (IYCF) information and counselling among primary healthcare (PHC) workers.

**Methods:**

A two-stage cluster sample was used to select health workers for training on IYCF in Ibadan, Nigeria. Baseline, immediate and 4-week post-training surveys were conducted to assess knowledge, attitudes and practices of health workers regarding IYCF. Paired *t*-tests were used to measure differences (*p* < 0.05) before and after the training.

**Results:**

A total of 124 health workers were trained on current global IYCF recommendations. Participants included community health extension workers (59.7%), nurses (27.4%), community health officers (11.3%), and pharmacy technicians (1.6%). Mean age was 41.8 ± 8.2 years and 95.2% were women. Knowledge of health workers regarding IYCF, particularly complementary feeding, was low at baseline but improved significantly following the training intervention. Attitudes and practices regarding provision of IYCF were suboptimal among health workers at the PHC facilities, but this improved with training.

**Conclusion:**

Health workers at the PHC level need regular retraining exercises to ensure effective counselling on IYCF.

## Introduction

The current global Infant and Young Child Feeding (IYCF) recommendation^1^ is that infants be exclusively breastfed for the first six months of life and thereafter receive safe and nutritionally adequate complementary foods while breastfeeding continues up to two years of age or beyond. There is abundance of evidence that optimal IYCF has both short-term and long-term benefits, particularly protecting children from morbidity and mortality^2^ and also ensures a child is protected from both under- and over-nutrition and their consequences later in life.^3^ It has been estimated that suboptimal breastfeeding results in an increased risk for mortality in the first two years of life with the number of child deaths attributed to this being 804 000 or 11.6% of all deaths in 2011 alone.^4^ Despite improvements in some countries, undesirable IYCF practices are still rife in many low- and middle-income countries, including Nigeria. The proportion of children under the age of six months that are exclusively breastfed decreased from 17% in 2003 to 13% in 2008^5^ and is currently at 17%, as shown in the 2013 Nigeria Demographic and Health Survey.^6^ Sixty-nine percent of children are predominantly breastfed (breast milk and only plain water or non-milk liquids such as juice, clear broth and other liquids); 13% of children under age two are bottle fed. Contrary to recommendations, 9% of children aged 0–1 month, 16% of children aged 2–3 months and 38% of children aged 4–5 months are given complementary foods in addition to breast milk.^6^

Researchers have documented some obstacles to IYCF practices in Nigeria to include low knowledge of EBF,^7^ maternal age whereby very young mothers were found to have inadequate knowledge on IYCF,^7,8^ and socio-cultural factors such as feeding infants herbal concoctions alongside breast milk.^9^ Others include mothers’ employment and proximity to infant,^8,10^ low level of education^10,11^ and low income.^10,11^ Mothers’ perceptions of insufficient milk supply,^12^ lack of confidence in mothers’ ability to breastfeed and problems with the infant latching or suckling as well as breast pain or soreness have also been documented.^13,14^ Some of these problems can be overcome through adequate antenatal information and preparation of expectant mothers.

Healthcare providers play an important educative and support role to mothers regarding lactation and infant feeding.^15,16^ The majority of health workers in primary healthcare (PHC) facilities in Nigeria are nurses, midwives, community health officers (CHOs), pharmacy technicians and community health extension workers (CHEWs). They are well positioned to provide adequate health information and counselling on infant feeding as a component of the services given to pregnant women during antenatal visits and may influence mothers’ attitudes and infant feeding practices. Health workers in PHCs are closer to the majority of the community population and are highly regarded by people within these communities, especially antenatal clinic attendees who look up to them for IYCF counselling, among others. They have been described as vanguards of information dissemination in Nigeria^17^; however, it has been observed that the IYCF information and counselling often provided at PHCs are often inadequate and needs to be strengthened.^18^ It is essential for health workers to have thorough and updated knowledge of IYCF recommendations to adequately provide correct information to mothers and promote optimal IYCF, yet existing opportunities and resources for retraining exercises or refresher courses for health workers in PHCs are few and far between. In addition, deliberate efforts should be made to incorporate continuing education workshops to better prepare health professionals for their role in providing tangible breastfeeding support at the primary care level.^18^

Based on the foregoing discussion, the study trained health workers in PHC facilities and evaluated the effect of the training on their knowledge, attitudes and provision of IYCF information and counselling to antenatal clinic attendees.

## Research methodology

### Study design

The study design was a before and after clinic-based intervention study.

### Study setting

The study was carried out in Ibadan, the capital city of Oyo State in South-western Nigeria. Ibadan comprises a total of 11 Local Government Areas (LGAs), of which five are classified as Ibadan municipality because of their characteristic high population density. All five LGAs were included in the study namely Ibadan North, Ibadan North East, Ibadan North West, Ibadan South East and Ibadan South West.

### Study population and sampling strategy

The following statistical formula was used to determine the sample size for a representative sample for a before and after pair of participants:

Number of pairs = [(*zα* + *zβ*)*σ_d_*]/*δ*^2^

where *zα* = 1.96 corresponding to a significance level of 5% for a two-tailed hypothesis,

*zβ* = 1.28, which is the probability of failing to reject a false null hypothesis (*β*) = 0.80, hence *zβ* = 1.28,

*σ_d_* = variance of the population being studied,

*δ* = the minimum desired effect that is worthwhile to be detected.

Number of pairs = [(1.96 + 1.28)16/5]^2^ = 108 health workers (increased to 120 to make room for 10% attrition). A total of 120 participants were thus needed to obtain a sample sufficiently powered to detect meaningful change from before to after the intervention.

The study population included health workers and antenatal clinic attendees in selected government-owned PHC facilities in all 5 LGAs of the Ibadan municipality. Such PHC facilities must be those that offer antenatal, delivery and postnatal services. To select the health workers, the study adopted a two-stage cluster sampling technique. In the first stage, two PHC facilities with the highest number of antenatal clinic attendees over the preceding 6-month period were purposively selected from each of the five LGAs, making a total of 10 PHCs. In the second stage, all health workers (nurses, nurse/midwives, CHOs, pharmacy technicians and CHEWs in each of the ten selected health facilities who had frequent contact with women who attended antenatal clinic were approached to participate in the study. A total of 124 health workers consented and participated in the study.

For each of the health workers, at least one client (antenatal clinic attendee) was randomly selected and also recruited for the study, after due informed consent was given. In all, 162 and 146 antenatal clinic attendees participated at baseline and at 4 weeks post-intervention, respectively.

### The intervention

For the intervention, a 2-day training session was designed and implemented in each PHC facility using lectures, interactive sessions, group work, quizzes, songs and role play. A training manual was developed for participants’ use during the training sessions. The training provided information and training on the following:

- Current IYCF recommendations
- Importance of one-on-one individualised counselling for antenatal attendees
- Good communication skills for one-on-one individualised counselling of antenatal attendees
- After the training sessions, posters and handbills were given to the health workers to assist them in their subsequent one-on-one counselling sessions with mothers.

### Data collection

The tools used for data collection during the surveys were two different structured questionnaires. The first questionnaire was used to collect data on health workers’ socio-demographic characteristics, job experience, knowledge of and attitudes to IYCF, attitudes towards communication with patients and reported practice of one-on-one individualised IYCF counselling. Knowledge of IYCF was assessed with a total of 20 questions on the fundamental understanding and core indicators of IYCF, as obtained from literature review of this subject matter. These knowledge questions were scored (correct answer = 1, incorrect answer = 0), with 20 being the highest score obtainable. The questionnaire was pilot-tested among 20 health workers outside the study location and evaluated for internal validity before the final questionnaire was approved for fieldwork.

Another structured (short) questionnaire was used to collect information from antenatal clinic attendees about their socio-demographic characteristics and whether they received individualised IYCF counselling from health workers in the health facilities where the study was carried out. This was carried out to complement the information reported by the health workers on their practice of one-on-one individualised IYCF counselling.

The health workers’ questionnaire was administered thrice: at baseline, immediately after the intervention (same day) and 4 weeks after intervention, while the second questionnaire was administered (twice) to antenatal clinic attendees at baseline and 4 weeks after the training intervention. Two female research assistants assisted the investigators in administering questionnaires to the mothers and collecting completed questionnaires from the health workers. They both had postgraduate degrees and were trained by the principal investigator for their roles.

### Data analysis

Data was entered and analysed using SPSS version 17.0. Chi-square test was used to test associations between categorical variables; while analysis of variance was used in comparing quantitative variables. Paired *t*-tests measured statistically significant differences (*p* < 0.05) before and after the training.

### Ethical considerations

Confidentiality was maintained and names were not used on data tools. Verbal informed consent was collected from all participants. No harm occurred to participants, and they were free to withdraw from the study at any point without any repercussion. Ethical approval was sought and obtained from the Oyo State Ministry of Health Ethical Review Board.

## Results

### Socio-demographic data

The socio-demographic characteristics of participants are shown in Table 1. A total of 124 participants were enrolled in the study, 123 (99.2%) of whom attended both training days, and 110 (88.7%) completed the 4-week post-training survey. Most participants were aged at least 40 years (59.3%), with a mean of 41.8 ± 8.2 years. Most participants (95.2%) were women, 90.3% were married and 76.6% were in a monogamous relationship. CHEWs (59.7%) formed the largest job category. Health workers had practiced their profession for between 1 and 35 years, with a mean of 16.2 ± 9.3 years.

**TABLE 1:** Socio-demographic characteristics of health workers.

Variable	Frequency (%)
**Age (in years)**	
20–39	50 (40.3)
40–59	73 (58.9)
No response	1 (0.8)
**Gender**	
Men	6 (4.8)
Women	118 (95.2)
**Local government area**	
Ibadan North	17 (13.7)
Ibadan North East	27 (21.8)
Ibadan North West	38 (30.6)
Ibadan South East	28 (22.6)
Ibadan South West	14 (11.3)
**Marital status**	
Single, never married	6 (4.8)
Married	112 (90.3)
Separated/divorced	2 (1.6)
Widowed	4 (3.2)
**Type of marriage**	
Monogamous	94 (75.8)
Polygamous	18 (14.5)
Not applicable	12 (9.7)
**Education**	
Nursing diploma	6 (4.8)
Midwifery diploma	14 (11.3)
Public Health Nursing diploma	7 (5.6)
Other diplomas	83 (66.9)
Tertiary education	14 (11.3)
**Job category**	
Nursing	34 (27.4)
Community health officer	14 (11.3)
Community health extension worker	74 (59.7)
Pharmacy technician	2 (1.6)
**Religion**	
Christianity	86 (69.4)
Islam	37 (29.8)
Traditionalist	1 (0.8)

*Source*: Authors own work

### Health workers’ knowledge of infant and young child feeding

Table 2 presents the responses of health workers to some of the knowledge questions on IYCF. At baseline, health workers correctly answered questions on early initiation (83.9%), exclusive breastfeeding (71.8%) and timely introduction of complementary feeding (79.0%). However, only 4.8% could list three advantages of breastfeeding for the baby, while 29.8% mentioned three advantages of breastfeeding to the mother. At immediate post-intervention there was an improvement, though not impressive, and this improvement waned at 4 weeks post-intervention. The proportion of participants who could state three breastfeeding difficulties (17.7%) also improved at immediate post-intervention (65.9%). This improvement was sustained at 4 weeks post-intervention, although there was a drop from the immediate post-intervention values but not as low as the baseline values. Participants’ knowledge was very poor at baseline with respect to the management of breastfeeding difficulties, as only 3.2% could mention three ways of management. This improved just slightly immediately after the intervention (7.3%), but dropped below baseline values after 4 weeks.

**TABLE 2:** Percentage of health workers who correctly answered (selected) knowledge questions.

Knowledge items	At baseline (*n* = 124)	Immediate post- intervention (*n* = 123)	4 weeks post- intervention (*n* = 110)	*p*-value
When baby should be offered breast (early initiation)	104 (83.9)	108 (87.8)	90 (81.8)	> 0.05
How baby under 6 months should be fed (exclusive breastfeeding)	89 (71.8)	90 (73.2)	79 (71.8)	> 0.05
Mentioned 3 advantages of breastfeeding to infant	6 (4.8)	26 (21.1)	10 (9.1)	< 0.05
Mentioned 3 advantages of breastfeeding to mother	37 (29.8)	76 (61.8)	49 (44.5)	< 0.05
Mentioned 3 common breastfeeding difficulties	22 (17.7)	81 (65.9)	56 (50.9)	< 0.05
Mentioned 3 ways of managing breastfeeding difficulties	4 (3.2)	9 (7.3)	3 (2.7)	> 0.05
Age baby should be introduced to first food (timely introduction of complementary feeding)	98 (79.0)	98 (79.7)	90 (81.8)	> 0.05
Correct consistency of complementary foods	30 (24.2)	30 (24.4)	42 (38.2)	< 0.05
No. of times baby should be fed (6–8 months)	20 (16.1)	77 (62.6)	44 (40.0)	< 0.05
No. of times baby should be fed (9–11 months)	40 (32.3)	98 (79.7)	67 (60.9)	< 0.05
No. of times baby should be fed (12–24 months)	49 (39.5)	74 (60.2)	59 (53.6)	< 0.05
Ever heard of 7 food group classification	68 (54.8)	113 (91.9)	100 (90.9)	< 0.05
Mentioned ≥ 4 food groups	2 (1.6)	83 (67.5)	27 (24.5)	< 0.05
Mentioned minimum number of food groups that should be in baby’s food	20 (16.1)	72 (58.5)	50 (45.5)	< 0.05
Mentioned 3 or more ways of improving complementary feeding	4 (3.2)	23 (18.7)	11 (10.0)	< 0.05

*Source*: Authors own work

The health workers performed poorly on questions about complementary feeding such as correct consistency (24.2%), classification of food groups in a baby’s diet (1.6%) and ways of improving complementary feeding (3.2%). The percentage of correct answers increased immediately after the intervention (24.4%, 67.5% and 18.7%, respectively), though this dropped at 4 weeks post-intervention. An exception to this trend were the questions on timely introduction of complementary feeding and correct consistency of complementary foods where the percentage of correct answers was lowest at baseline and highest at the 4-week post-intervention stage. However, overall, the increase in mean knowledge score at baseline, immediate post-intervention and 4 weeks post-intervention (18.9 ± 2.2, 20.1 ± 2.8, 20.0 ± 2.5) was statistically significant (*P* = 0.0001).

### Health workers’ attitudes to infant and young child feeding

As presented in Table 3, results for health workers’ attitude to infant feeding at the baseline show that although most health workers agreed to the adequacy of breast milk (alone) in the first 6 months of life (90.3%), fewer (85.5%) agreed to the feasibility of exclusive breastfeeding. Few (12.9%) agreed that mothers with HIV infection should breastfeed while about half (55.6%) believed that complementary foods should be introduced at whatever age the infant is ready. At immediate post-intervention and 4 weeks after, more health workers agreed to sufficiency of breast milk in the first 6 months (96.7%; 98.2%), feasibility of exclusive breastfeeding (92.6%; 96.4%) and breastfeeding in HIV (47.2%; 43.6%). Compared to baseline values, there was no significant difference in attitudes regarding the introduction of complementary feeding whenever the child is ready (49.6%, 48.2%).

**TABLE 3:** Health workers attitudes to infant and young child feeding.

Attitude items	Baseline	Immediate post- intervention	4 weeks post- intervention	*p*-value
I agree that breast milk alone is enough food for a baby in the first 6 months of life	112 (90.3)	119 (96.7)	108 (98.2)	< 0.05
I agree that it is possible for women in this community to breastfeed exclusively for 6 months	106 (85.5)	114 (92.7)	106 (96.4)	< 0.05
I think a mother with HIV should breastfeed her baby (in a resource-poor setting like ours)	16 (12.9)	58 (47.2)	48 (43.6)	< 0.05
I believe that complementary foods should be introduced to an infant any time the child is ready.	69 (55.6)	61 (49.6)	53 (48.2)	< 0.05

*Source:* Authors own work

Health workers’ attitudes to communicating with mothers during one-on-one individualised IYCF counselling were also investigated. The proportion of health workers who agreed they should treat mothers respectfully at baseline (94.4%) increased at immediate and 4 weeks post-intervention (99.2%, and 100.0%). At baseline, most workers reported they would give practical advice (91.9%). This proportion increased at immediate and 4 weeks post-intervention (99.2%, 98.2%). Also at baseline, most health workers would avoid technical language (83.1%), be patient (87.1%) and address mothers by name (79.9%) when dealing with them. These percentages increased at immediate and 4 weeks post-intervention as more health workers would avoid technical language (97.5%, 96.3%), be patient (95.2%, 96.3%) and address the mothers by name (95.2%, 96.3%). However, the differences observed at immediate and 4 weeks post-intervention were not statistically significant.

### Health workers’ practice of one-on-one individualised infant and young child feeding counselling

Before the intervention, 79.0% reported that they provided practical tips on breastfeeding, 79.0% gave counselling on HIV and infant feeding options, while only 45.7% gave any information on complementary feeding. At 4 weeks post-intervention, the proportion of health workers who reported giving practical tips on breastfeeding increased (98.2%), and the same holds true for the proportion who gave counselling on HIV and infant feeding options (91.0%) and information on complementary feeding (88.2%).

However, when asked about whether they had received one-on-one counselling from health workers, the responses of the antenatal clinic attendees differed from those of the health workers. At baseline and 4 weeks after the training intervention, interviews were held with 162 (mean age: 27.3 ± 5.6 years) and 146 (mean age: 26.9 ± 5.5 years) antenatal clinic attendees, respectively. Of the 162 mothers surveyed at baseline, 131 (80.9%) women had made at least three antenatal clinic visits, 9.3% received counselling on breastfeeding, 8.0% received counselling on HIV and infant feeding options while 7.4% received counselling on complementary feeding. Similarly, for the group of 146 women interviewed 4 weeks after the training intervention for health workers, although 110 (75.3%) had made at least three antenatal appointments, only 6.2% reported that they received counselling on breastfeeding, 5.5% received counselling on HIV and infant feeding, whereas 8.0% had received complementary feeding information. The proportion of women who reported receiving individualised one-on-one IYCF counselling sessions did not improve significantly following the training intervention for health workers (Fisher’s exact test: *p* = 0.17).

## Discussion

Generally at baseline, the health workers in the study displayed better knowledge about breastfeeding than complementary feeding. However, it is interesting to note that health workers did better in the ‘yes or no’ knowledge questions than in questions that required them to list the answers. This was particularly shown in the questions regarding breastfeeding difficulties and their management. This finding is in agreement with that of Utoo and others,^17^ who found in their study among selected health workers in South-South Nigeria that a majority of the respondents could not mention three advantages of breastfeeding. An earlier study also in Nigeria also reported that healthcare providers lack adequate breastfeeding knowledge and may show unhelpful attitudes and practices.^19^

A possible explanation for the pattern of observation in the study may be that health workers had general knowledge of the recommendations for infant feeding, especially exclusive breastfeeding, but lacked in-depth knowledge of specific aspects. Such knowledge hiatus should be the focus of training and re-training activities for health workers who provide health information and counselling on IYCF as a component of the services given to pregnant women during antenatal visits. These contacts should be leveraged on to influence mothers’ intentions and support their practice of optimal IYCF.

In the study, the prevalent positive attitudes towards adequacy and feasibility of exclusive breastfeeding, complemented by positive attitudes to communicating with mothers are commendable and may serve as a springboard to promote IYCF in the community. Yet, as observed, some negative attitudes persisted regarding HIV and infant feeding, as well as for complementary feeding. This goes hand in hand with observed poor knowledge of health workers in these aspects both before and after intervention, suggesting deeply rooted influences (possibly socio-cultural) that remain among health workers and shape their opinions despite previous medical or public health training and re-training (as in the intervention study). Behaviour change communication in IYCF must start from health workers; hence, training and re-training of this group should involve strategies that embrace activities that can supplement the usual didactic method of teaching clinical facts and imparting desired medical skills. Such pedagogies should not only enhance cognitive learning and skills acquisition but also be very effective to bring about behaviour change in the learner.

Regarding practice of one-on-one counselling sessions with mothers, a large proportion of health workers claimed to practice this at baseline, and this increased even further at 4 weeks post-intervention. However, it is interesting to note that the interviews with the mothers did not agree with this finding as they reported receiving such counselling on IYCF only rarely. It is possible that the health workers over-rated their interaction with antenatal clinic attendees. It is also possible that some health workers were unable to distinguish between group health talks and one-on-one counselling sessions with antenatal clinic attendees. Another possibility is that this is a reflection of social desirability bias, as the health workers may have responded in the way they felt they were expected to.

On the whole, the outcomes of the training intervention were positive. Generally, knowledge and attitudes improved immediately following the training intervention. This is similar to other studies where nutrition education and training of health workers improved personal knowledge, counselling practices and enhanced the communication skills and performance of health workers.^20,21^ A systematic review^22^ also showed that nutrition training for health workers can improve feeding frequency, energy intake and dietary diversity of children aged 6 months to 2 years. However, in the present study, the improvements had fallen again by the 4-week post-training visit, though to a level not as low as the baseline level. Longer intervention duration may have been more effective because of a longer exposure of participants to training. The short (2-day) duration of training in this study is thus a possible limitation of study.

## Conclusion

In-depth knowledge of IYCF was lower than expected in the study population at baseline but attitudes were generally positive. Although the training intervention improved knowledge, attitudes and practice of health workers regarding IYCF, these improvements need to be strengthened and sustained. Innovative in-service training and regular retraining exercises at the local government level can help to improve the status quo in this regard.

## References

[1] World Health Organization Infant and young child feeding fact sheet N342 [homepage on the Internet]. [Cited 2015 Dec 28]. Available from http://www.who.int/mediacentre/factsheets/fs342/en/#

[2] Robert EB, Cesar GV, Walker SP, et al Maternal and child undernutrition and overweight in low-income and middle-income countries. Lancet. 2013;382:372–375. http://dx.doi.org/10.1016/S0140-6736(13)60937-X 23746772 10.1016/S0140-6736(13)60937-X

[3] United Nations Children‘s Fund Infant and Young Child Feeding Programming Guide. UNICEF, New York 2011.

[4] Robert EB, Lindsay HA, Zulfiqar AB, et al Maternal and child undernutrition: global and regional exposures and health consequences. Lancet. 2008;371:243–246. http://dx.doi.org/10.1016/S0140-6736(07)61690-0 18207566 10.1016/S0140-6736(07)61690-0

[5] National Population Commission (NPC) [Nigeria], ICF Macro Nigeria Demographic and Health Survey. Abuja, Nigeria: National Population Commission and ICF Macro; 2008.

[6] National Population Commission (NPC) [Nigeria], ICF International Nigeria Demographic and Health Survey. Abuja, Nigeria: NPC and ICF International; 2013.

[7] Ojofeitimi EO, Owolabi OO, Eni-Olorunda JT, Adesina OF, Esimai OA Promotion of exclusive breastfeeding (EBF): the need to focus on the adolescents. Nutr Health 2001;15:55–62. http://dx.doi.org/10.1177/026010600101500107 11403374 10.1177/026010600101500107

[8] Lawoyin TO, Olawuyi JF, Onadeko MO Factors associated with exclusive breastfeeding in Ibadan, Nigeria. J Hum Lact. 2001;17:321–325. http://dx.doi.org/10.1177/089033440101700406 11847901 10.1177/089033440101700406

[9] Agunbiade MO, Ogunleye VO Constraints to exclusive breastfeeding practices among breastfeeding mothers in Southwest Nigeria: implication for scaling up [homepage on the Internet].[Cited 2014 Aug 24]. Int Br J. 2012;7:5 Available from http://www.internationalbreastfeedingjournal.com/content/7/1/5 10.1186/1746-4358-7-5 PMC3359265 22524566

[10] Salami LI Factors influencing breastfeeding practices in Edo State, Nigeria. Ajfand. 2006;6:1–12.

[11] Agho KE, Dibley MJ, Odiase JI, Ogbonmwan SM Determinants of exclusive breastfeeding in Nigeria [homepage on the Internet]. [Cited 2013 Oct 10] BMC Pregnancy Childbirth. 2011;11:2 Available from http://www.biomedcentral.com/1471-2393/11/2 21219659 10.1186/1471-2393-11-2 PMC3025918

[12] Davies-Adetugbo A Sociocultural factors and the promotion of exclusive breastfeeding in rural Yoruba communities of Osun State, Nigeria. Soc Sci Med. 1997;45:113–125. 9203276 10.1016/s0277-9536(96)00320-6

[13] Ukegbu AU, Ukegbu PO, Onyeonoro UU, et al Determinants of breastfeeding patterns among mothers in Anambra State, Nigeria. South Afr J Child Health. 2011;l5:1–12. 23120962

[14] Sholeye OO, Abosede OA, Salako AA Exclusive breastfeeding and its associated factors among mothers in Sagamu, Southwest Nigeria. J Health Sci. 2015;5:25–31. http://dx.doi.org/10.5923/j.health.20150502.01

[15] Dykes F The education of health practitioners supporting breastfeeding women: time for critical reflection. Matern Child Nutr. 2006;2:204–216. 16999766 10.1111/j.1740-8709.2006.00071.x PMC6860707

[16] Wallace LM, Kasmala-Anderson J Training needs survey of midwives, health visitors and voluntary-sector breastfeeding support staff in England. Matern Child Nutr. 2007;325–339. 10.1111/j.1740-8709.2007.00079.x PMC6860884 17238933

[17] Utoo BT, Ochejele S, Obulu MA, Utoo PM Breastfeeding knowledge and attitudes amongst health workers in a health care facility in south-south Nigeria: the need for middle level health manpower development. Mother Child Health. 2012;9:1–5. http://dx.doi.org/10.4303/cmch/235565

[18] Olaolorun FM, Lawoyin TO Health workers’ support for breastfeeding in Ibadan, Nigeria. J Hum Lact. 2006;22:188–194. 16684907 10.1177/0890334406287148

[19] Owoaje ET, Oyemade A, Kolude OO Previous BFHI training and nurses’ knowledge, attitudes and practices regarding exclusive breastfeeding. Afr J Med Med Sci. 2002;31:137–140. 12518909

[20] Khoury AJ, Hinton A, Mitra A, et al Improving breastfeeding knowledge, attitudes and practices of WIC Clinic Staff. Public Health Rep. 2002;117:453–462. 12500962 10.1016/S0033-3549(04)50186-3 PMC1497475

[21] Shakila Z, Ashraf RN, Martines J Training in complementary feeding counselling of healthcare workers and its influence on maternal behaviours and child growth: a cluster randomized controlled trial in Lahore, Pakistan. J Health Popul Nut. 2008;26:210–222. PMC2740673 18686554

[22] Sunguya BF, Poudel KC, Mlunde LB, et al Effectiveness of nutrition training of health workers toward improving caregivers’ feeding practices for children aged six months to two years: a systematic review. Nutr J. 2013;12:66. 23688174 10.1186/1475-2891-12-66 PMC3668136

